# Initial genome sequencing of the sugarcane CP 96-1252 complex hybrid

**DOI:** 10.12688/f1000research.11629.1

**Published:** 2017-05-17

**Authors:** Jason R. Miller, Kari A. Dilley, Derek M. Harkins, Manolito G. Torralba, Kelvin J. Moncera, Karen Beeri, Karrie Goglin, Timothy B. Stockwell, Granger G. Sutton, Reed S. Shabman

**Affiliations:** 1 J. Craig Venter Institute, Rockville, MD, 20850, USA; 2 J. Craig Venter Institute, La Jolla, CA, 92037, USA

**Keywords:** Sugarcane genome, DNA sequencing, sequencing reads

## Abstract

The CP 96-1252 cultivar of sugarcane is a complex hybrid of commercial importance. DNA was extracted from lab-grown leaf tissue and sequenced. The raw Illumina DNA sequencing results provide 101 Gbp of genome sequence reads. The dataset is available from https://www.ncbi.nlm.nih.gov/bioproject/PRJNA345486/.

## Introduction

Sugarcane is an important crop for food and energy production. The genomes of modern cultivars are hybrids of species that are themselves polyploid; see for example ( Vilela *et al.*, 2017). Selected genomic BAC sequences have been sequenced and assembled ( de Setta *et al.*, 2014) ( Okura *et al.*, 2016). Chloroplast and mitochondrial genomes have been published ( Asano *et al.*, 2004) ( Shearman *et al.*, 2016), as have several transcriptomes ( Cardoso-Silva *et al.*, 2014). Whole genome sequence assemblies have not been published. CP 96-1252 is the top commercial sugarcane cultivar in Florida, USA ( Sandhu & Davidson, 2016). CP 96-1252 was developed by USDA-ARS, the University of Florida, and the Florida Sugar Cane League and released to growers in 2003. CP 96-1252 is a complex hybrid of *Saccharum officinarum* L., *S. barberi* Jeswiet, *S. spontaneum* L., and *S. sinense* Roxb. amend. Jeswiet ( Edmé *et al.*, 2005). Toward better understanding of this cultivar through its genome sequence, DNA reads were generated and made public.

## Methods

Using lab-grown plantlets, kindly provided by USDA, 14 g of tissue was harvested from the leaves of *Saccharum* hybrid cultivar CP 96-1252 (Reg. no CV-120, PI 634935, NCBI taxon ID 1983727). DNA was extracted from purified plant nuclei at Amplicon Express (Pullman, WA, USA). Separately, DNA was extracted from whole cells at JCVI (Rockville, MD, USA) using a Qiagen Plant DNA isolation kit. Extracted DNA was fragmented and size selected on the Blue Pippin (Sage Scientific) prior to library construction to ensure a 260 bp insert size. Standard Illumina PE libraries were generated using the NEBNext kit (NEB). Libraries were size selected, QC’d and quantified by qPCR prior to sequencing. Barcode BS78 AGCCATGC was used for the nuclei prep library and barcode BS79 AGGCTAAC was used for the cell prep library. The libraries were generated and sequenced at the JCVI sequencing core in La Jolla, CA, USA. To test for bacterial contamination, both DNA samples plus negative controls were used to generate amplicon libraries targeting the V4 16S region followed by Illumina MiSeq sequencing. These reads were processed by a pipeline using usearch version 8.1.1.1861 for clustering ( Edgar, 2017), mothur version 1.36.1 for taxonomic classification ( Schloss *et al.*, 2011), and the SILVA SSURef NR99 123 database for reference ( Quast *et al.*, 2013). Hits to chloroplast and mitochondria were observed as expected, but bacteria were virtually absent and similar to controls.

An Illumina NextSeq 500 instrument was used to generate paired 150 bp shotgun reads. Run #1 applied the Illumina High Output kit to libraries BS78 and BS79. Run #1 instrument metrics were: 1.8 pM pool loaded, 1% PhiX spike-in with 1.8% aligned, cluster density 138 K/mm ^2^, 96% pass filter, and 106 Gbp in 345 M PE reads. Barcode analysis indicated 46% BS78 and 49% BS79. Run #2 applied the Illumina High Output kit to library BS78 only. Run #2 metrics were: 1.8 pM pool loaded, 1% PhiX spike-in with 1% aligned, and 110 Gbp in 360 M PE reads. The resulting FASTQ files contained 101 Gbp in 161 M pairs from BS78 run #1, 169 M pairs from BS79 run #1, and 341 M pairs from BS78 run #2.

## Dataset validation

To confirm sugarcane origin of the DNA, the run #1 reads were mapped to available BACs, namely the 608 Kbp of R570 BACs (GenBank accessions KF184657.1 to KF184973.1 ( de Setta *et al.*, 2014)). Reads were mapped with bowtie2 ( Langmead & Salzberg, 2012) version 2.2.5 with options “-p 4 --no-unal --no-mixed --no-discordant --end-to-end --fast”. Both sequencing libraries demonstrated concordant pair mapping rates of 4.1% unique, 27% repeat, and 69% unmapped. Genome coverage analysis was inconclusive; the K-mer frequency distribution computed by Jellyfish ( Marçais & Kingsford, 2011) version 2.2.4, with K=17 showed no peak above 1X coverage.

## Data availability

The data referenced by this article are under copyright with the following copyright statement: Copyright: © 2017 Miller JR et al.

Data associated with the article are available under the terms of the Creative Commons Zero "No rights reserved" data waiver (CC0 1.0 Public domain dedication). 

The data are available at NCBI SRA under BioProject PRJNA345486, Study SRP091668. Amplified reads from BS78 and BS79 have respective accessions SRR5500242 and SRR5500243. Genomic reads from BS78 have accessions are SRR5500246 and SRR5500247. Genomic reads from BS79 have accession SRR5500249.

## References

[ref-1] Asano T Tsudzuki T Takahashi S: Complete nucleotide sequence of the sugarcane ( *Saccharum officinarum*) chloroplast genome: a comparative analysis of four monocot chloroplast genomes. *DNA Res.* 2004;11(2):93–99. 10.1093/dnares/11.2.93 15449542

[ref-2] Cardoso-Silva CB Costa EA Mancini MC: *De novo* assembly and transcriptome analysis of contrasting sugarcane varieties. *PLoS One.* 2014;9(2):e88462. 10.1371/journal.pone.0088462 24523899 PMC3921171

[ref-3] de Setta N Monteiro-Vitorello CB Metcalfe CJ: Building the sugarcane genome for biotechnology and identifying evolutionary trends. *BMC Genomics.* 2014;15(1):540. 10.1186/1471-2164-15-540 24984568 PMC4122759

[ref-4] Edgar RC: SEARCH_16S: A new algorithm for identifying 16S ribosomal RNA genes in contigs and chromosomes. *bioRxiv.* 2017; 124131. 10.1101/124131

[ref-5] Edmé S Tai P Glaz B: Registration of 'CP 96-1252' sugarcane. *Crop Sci.* 2005;45(1):423–424. 10.2135/cropsci2005.0423

[ref-6] Langmead B Salzberg SL: Fast gapped-read alignment with Bowtie 2. *Nat Methods.* 2012;9(4):357–359. 10.1038/nmeth.1923 22388286 PMC3322381

[ref-7] Marçais G Kingsford C: A fast, lock-free approach for efficient parallel counting of occurrences of *k*-mers. *Bioinformatics.* 2011;27(6):764–770. 10.1093/bioinformatics/btr011 21217122 PMC3051319

[ref-8] Okura VK de Souza RS de Siqueira Tada SF: BAC-Pool Sequencing and Assembly of 19 Mb of the Complex Sugarcane Genome. *Front Plant Sci.* 2016;7:342. 10.3389/fpls.2016.00342 27047520 PMC4804495

[ref-9] Quast C Pruesse E Yilmaz P: The SILVA ribosomal RNA gene database project: improved data processing and web-based tools. *Nucleic Acids Res.* 2013;41(Database issue):D590–596. 10.1093/nar/gks1219 23193283 PMC3531112

[ref-10] Sandhu H Davidson W: Sugarcane Cultivars Descriptive Fact Sheet: CP 96-1252, CP 01-1372, and CP 00-1101.2016 Reference Source

[ref-11] Schloss PD Gevers D Westcott SL: Reducing the effects of PCR amplification and sequencing artifacts on 16S rRNA-based studies. *PLoS One.* 2011;6(12):e27310. 10.1371/journal.pone.0027310 22194782 PMC3237409

[ref-12] Shearman JR Sonthirod C Naktang C: The two chromosomes of the mitochondrial genome of a sugarcane cultivar: assembly and recombination analysis using long PacBio reads. *Sci Rep.* 2016;6: 31533. 10.1038/srep31533 27530092 PMC4987617

[ref-13] Vilela MM Del Bem LE Van Sluys MA: Analysis of Three Sugarcane Homo/Homeologous Regions Suggests Independent Polyploidization Events of *Saccharum officinarum* and *Saccharum spontaneum*. *Genome Biol Evol.* 2017;9(2):266–278. 10.1093/gbe/evw293 28082603 PMC5381655

