# Supplementary material for: Initial genome sequencing of the sugarcane CP 96-1252 complex hybrid
Source: F1000Res. 2017 May 17;6:688. [Version 1] doi: 10.12688/f1000research.11629.1 (PMC5497815; doi:10.12688/f1000research.11629.1)
Supplement: Peer review history

# Peer review: Referee response for version 1

**DOI:** 10.5256/f1000research.12560.r23843

**Published:** 2017-06-29

**Authors:** Jeremy R. Shearman

**Affiliations:** 1 National Center for Genetic Engineering and Biotechnology (BIOTEC), Khlong Luang, Thailand

The manuscript describes the generation of whole genome shotgun sequence data from two separate DNA preparation methods. The methods for data generation are clearly described and the sample that was used has ample information about its origins publicly available and referenced. This dataset will be useful for SNP discovery and comparative genomics of sugarcane cultivars.

I have read this submission. I believe that I have an appropriate level of expertise to confirm that it is of an acceptable scientific standard.

# Peer review: Referee response for version 1

**DOI:** 10.5256/f1000research.12560.r22855

**Published:** 2017-05-30

**Authors:** Paulo Arruda

**Affiliations:** 1 Center of Molecular Biology and Genetic Engineering (CBMEG), State University of Campinas (UNICAMP), Campinas, Brazil

The data note reported was produced by 150bp paired-end Illumina sequencing of genomic DNA prepared from the sugarcane variety CP-96-1252. A raw data set of 101 Gbps was generated and made public available.The authors did not present assemblage data which would be useful for the research community interested in sugarcane genomics. Sequence coverage was not stimated but it seems to be under 1X.  

 Sugarcane commercial varieties are hybrids between Saccharum officinarum and Saccharum spontaneum. These two parents are highly complex polyploids with ploidy varying from 8-12. In general, the hybrids conserve ~75% of the S. officinarum and 15% of S. spontaneum intact. Around 10% of the hybrid genome are chromosomal recombinants between the two species. This complex situation makes it very difficult assembling large non-chimeric contigs especially using short insert shotgun sequencing. 

 The high quality data set presented in this data note is of value for those interested in recover short gene regions of interest. Because sugarcane genome sequencing dataset is very scarse I recommend the publication of the note presented here as a source of genome data for the sugarcane community.

I have read this submission. I believe that I have an appropriate level of expertise to confirm that it is of an acceptable scientific standard.
